# Supplementary material for: A new limestone-dwelling species of Micryletta (Amphibia: Anura: Microhylidae) from northern Vietnam
Source: PeerJ. 2018 Oct 4;6:e5771. doi: 10.7717/peerj.5771 (PMC6174876; doi:10.7717/peerj.5771)
Supplement: Supplemental Information 1 — GenBank AN–GenBank accession number. [file peerj-06-5771-s001.docx]

| **No.** | **Species** | **GenBank A.N.** | **Specimen ID** | **Country** | **Locality** |
| --- | --- | --- | --- | --- | --- |
| **1** | *Micryletta* cf. *inornata* | AB611968 | KUHE-35133 | Laos | no locality |
| **2** | *Micryletta* cf. *inornata* | KC180027 | K-3246 | Laos | Luangprabang |
| **3** | *Micryletta* cf. *inornata* | AB598341 | KUHE-20497 | Thailand | Phrae, Mae Yom |
| **4** | *Micryletta* cf. *inornata* | KC179997 | FMNH-255121 | Laos | Khammouan |
| **5** | *Micryletta* cf. *inornata* | AF215375 | — | Thailand | no locality |
| **6** | *Micryletta* cf. *inornata* | AF285206 | TZ9892 | Vietnam | Ha Tinh, Ke Go |
| **7** | *Micryletta* cf. *inornata* | AF285207 | TZ9892 | Vietnam | Ha Tinh, Ke Go |
| **8** | *Micryletta* cf. *inornata* | MH879843 | ZMMU NAP-3352-1 | Vietnam | Hai Phong, Cat Ba N.P. |
| **9** | *Micryletta* cf. *inornata* | MH879844 | ZMMU NAP-3352-2 | Vietnam | Hai Phong, Cat Ba N.P. |
| **10** | *Micryletta* cf. *inornata* | MH879845 | ZMMU NAP-3580 | Vietnam | Hai Phong, Cat Ba N.P. |
| **11** | *Micryletta* cf. *inornata* | MH879840 | DTU 310 | Vietnam | Ninh Binh, Cuc Phuong N.P. |
| **12** | *Micryletta* cf. *inornata* | MH879841 | DTU 311 | Vietnam | Ninh Binh, Cuc Phuong N.P. |
| **13** | *Micryletta* cf. *inornata* | MH879842 | DTU 312 | Vietnam | Ninh Binh, Cuc Phuong N.P. |
| **14** | *Micryletta steinegeri* | AB634696 | KUHE-35937 | Taiwan | Yunling |
| **15** | *Micryletta erythropoda* | AB634695 | KUHE-23858 | Thailand | Ranong |
| **16** | *Micryletta erythropoda* | MH756146 | ZMMU A4721-1533 | Vietnam | Dong Nai, Ma Da (Vinh Cuu) N.R. |
| **17** | *Micryletta erythropoda* | MH756147 | ZMMU A4721-1542 | Vietnam | Dong Nai, Ma Da (Vinh Cuu) N.R. |
| **18** | *Micryletta nigromaculata* **sp. nov.** | MH756148 | ZMMU A5947 | Vietnam | Hai Phong, Cat Ba N.P. |
| **19** | *Micryletta nigromaculata* **sp. nov.** | MH756149 | ZMMU A5937 | Vietnam | Hai Phong, Cat Ba N.P. |
| **20** | *Micryletta nigromaculata* **sp. nov.** | MH756150 | ZMMU A5934 | Vietnam | Hai Phong, Cat Ba N.P. |
| **21** | *Micryletta nigromaculata* **sp. nov.** | MH756152 | ZMMU A5940 | Vietnam | Hai Phong, Cat Ba N.P. |
| **22** | *Micryletta nigromaculata* **sp. nov.** | MH756151 | ZMMU A5946 | Vietnam | Hai Phong, Cat Ba N.P. |
| **23** | *Micryletta nigromaculata* **sp. nov.** | MH756153 | ZMMU A5942 | Vietnam | Hai Phong, Cat Ba N.P. |
| **24** | *Micryletta nigromaculata* **sp. nov.** | MH756154 | DTU 301 | Vietnam | Ninh Binh, Cuc Phuong N.P. |
| **25** | *Micryletta nigromaculata* **sp. nov.** | MH756155 | DTU 303 | Vietnam | Ninh Binh, Cuc Phuong N.P. |
| **26** | *Micryletta nigromaculata* **sp. nov.** | MH756154 | DTU 304 | Vietnam | Ninh Binh, Cuc Phuong N.P. |
| **27** | *Microhyla heymonsi* | AB598312 | KUHE23856 | Thailand | Ranong |
| **28** | *Microhyla fissipes* | AB201174 | KUHE32943 | China | Anhui, Huang Shan |
| **29** | *Microhyla annectens* | AB634600 | KUHE53373 | Malaysia | Selangor, Genting |
| **30** | *Glyphoglossus yunnanensis* | AB634626 | KUHE44148 | China | no locality |
| **31** | *Glyphoglossus guttulatus* | AB634627 | KUHE35163 | Thailand | Kanchanaburi, Pilok |
| **32** | *Glyphoglossus molossus* | AB201182 | KUHE35182 | Thailand | Tak, Barrntak |
| **33** | *Kaloula picta* | AB634628 | KUHEUN | Philippines | no locality |
| **34** | *Kaloula baleata* | AB634629 | KUHE32313 | Indonesia | Sumba |
| **35** | *Kaloula borealis* | AB634630 | KUHE33139 | Korea | Cheju |
| **36** | *Kaloula mediolineata* | AB634631 | KUHE35178 | Thailand | Tak, Barrntak |
| **37** | *Kaloula pulchra* | AB634632 | KUHE22206 | Thailand | Nong Khai |
| **38** | *Uperodon taprobanicus* | AB634633 | KUHE37252 | Sri Lanka | no locality |
| **39** | *Metaphrynella pollicaris* | AB634634 | KUZ-21655 | Malaysia | Pahang, Fraser’s Hill |
| **40** | *Metaphrynella sundana* | AB634635 | BOR8191 | Malaysia | Sabah, Crocker |
| **41** | *Phrynella pulchra* | AB634636 | UKMHC-820 | Malaysia | Trengganu, Hulu Trengganu |
| **42** | *Chaperina fusca* | AB598318 | BORN8478 | Malaysia | Sabah, Crocker |
| **43** | *Kalophrynus interlineatus* | AB634640 | KUHE33787 | Myanmar | Chatthin |
